# A Qualitative Study of CMC Caregivers’ Perspectives about their Emotional Well-Being

**DOI:** 10.1016/j.jpainsymman.2025.03.028

**Published:** 2025-04-03

**Authors:** Justin A. Yu, Amy Porter, Jori Bogetz, Mikhaila Layshock, Lydia McLachlan, Sydney Weill, Joseph G. Winger, Maya I. Ragavan, Abby Rosenberg, Amy Houtrow, Robert Noll, Yael Schenker

**Affiliations:** Department of Pediatrics (PaRC), Palliative Research Center (J.A.Y), University of Pittsburgh; Division of Palliative and Supportive Care, University of Pittsburgh School of Medicine and UPMC Children’s Hospital, Pittsburgh, PA, USA; Department of Pediatrics (A.P.), Division of Supportive and Palliative Care, Mass General for Children, Massachusetts General Hospital, Harvard Medical School, Boston, MA, USA; Department of Pediatrics (J.B.), Division of Bioethics and Palliative Care, University of Washington School of Medicine; Treuman Katz Center for Pediatric Bioethics and Palliative Care, Center for Clinical and Translational Research, Seattle Children’s Research Institute, Seattle, WA, USA; University of Pittsburgh School of Medicine (M.L.), Pittsburgh, PA, USA; Case Western Medical School (L.M.), Cleveland, OH, USA; University of Pittsburgh Medical Center (S.W.), Pittsburgh, PA, USA; Department of Psychiatry and Behavioral Sciences (J.G.W.), Duke University School of Medicine, Durham, NC, USA; Division of General Academic Pediatrics (M.I.R.), University of Pittsburgh School of Medicine and UPMC Children’s Hospital, Pittsburgh, PA, USA; Department of Pediatrics (A.R.), Division of Psychosocial Oncology and Palliative Care, Dana Farber Cancer Institute; Boston Children’s Hospital; Department of Pediatrics, Harvard Medical School, Boston, MA, USA; Department of Physical Medicine & Rehabilitation (A.H.), University of Pittsburgh School of Medicine, Pittsburgh, PA, USA; University of Pittsburgh (R.N.), Emeritus, Pittsburgh, PA, USA; Division of General Internal Medicine (Y.S.), Palliative Research Center (PaRC), University of Pittsburgh; Section of Palliative Care and Medical Ethics, University of Pittsburgh School of Medicine, Pittsburgh, PA, USA

**Keywords:** Children with medical complexity, Family caregivers, Emotional well-being, Pediatric palliative care

## Abstract

**Objective.:**

To better understand the perspectives of family caregivers’ of children with medical complexity (CMC) about their emotional well-being.

**Methods.:**

We conducted a qualitative study of family caregivers of CMC receiving medical care at an academic children’s hospital in western Pennsylvania. Participants completed one-on-one, in-depth, semi-structured interviews examining their perspectives about their emotional well-being in the context of CMC caregiving. We used a constant-comparative and inductive approach to analyze deidentified transcripts and identify emergent themes.

**Results.:**

We interviewed 19 participants (17 [90%] female; 14 [74%] White; mean age 43 years) from March to December 2022. Participant’s children were 11 years old on average (range 2–20 years), utilized medical technology (19 [100%]), and lived with chronic conditions most commonly affecting the neurologic (19 [100%]), gastrointestinal (19 [100%]), and respiratory (16 [84%]) organ systems. Three emergent themes reflected how CMC caregivers’ emotional well-being exists on a dynamic spectrum: 1) deep reward vs. sadness, 2) increased and decreased control of one’s emotions, and 3) psychological strength vs. exhaustion and defeat. Participants reported experiencing these seemingly conflicting emotions either concurrently or oscillating between them.

**Conclusion.:**

Family caregivers of CMC report both strongly positive and negative changes to their emotional well-being which are often dynamic and in tension. Our findings can be used to inform the development and implementation of future clinical and research efforts aiming to improve the emotional well-being of this critically important caregiver population.

## Introduction

Children with medical complexity (CMC) live with chronic health conditions and functional limitations resulting in substantial care needs.^1^ CMC are one of the fastest growing pediatric populations (now >1 million US children) and account for over one-third of annual pediatric health care spending.^2,3^ As most CMC live at home, their family caregivers have emerged as essential partners in the operations of pediatric health systems.^3^ CMC caregivers are responsible for constant monitoring, demanding care regimens, and complex care coordination in the context of unpredictable daily schedules, uncertainty about the future, fragmented health systems, and under-resourced home-based services.^4-6^

As family caregiver emotional well-being is directly linked to child health and development, our field’s ability to promote CMC caregivers’ emotional well-being is a national priority with significant public health implications.^7,8^ However, a growing body of evidence indicates that CMC caregivers’ experience reduced emotional well-being, which consists of interrelated concepts such as mental health, quality of life, positive and negative emotions, and life satisfaction.^9-11^ Recent work demonstrates that CMC caregivers report poor over-all mental health, reduced mental health-related quality of life, clinically significant levels of emotional distress, poor parental coping and frequent parental aggravation, and reduced family functioning.^12^

However, our understanding about CMC caregivers’ emotional well-being remains inadequate as in-depth and balanced examination of caregivers’ perspectives about their emotional well-being is lacking. Prior research has largely examined emotional well-being using general and/or unidimensional measures (e.g., global mental health) and been negatively oriented, thus ignoring potentially beneficial aspects of caregiving.^13,14^ Therefore, we conducted a qualitative study among CMC family caregivers to gain a more holistic understanding of how they conceptualize emotional well-being and perceive it changes in the context of their unique caregiving experience.

## Methods

This was part of a qualitative study examining the experiences of CMC caregivers in coping with caregiving-related stress. This study followed the consolidated criteria for reporting qualitative research guidelines (see Supplement) and was approved by the University of Pittsburgh’s Institutional Review Board.^15^

Participants were family caregivers of CMC receiving medical care at an academic pediatric hospital in western Pennsylvania. We defined medical complexity using our institution’s Complex Care Center’s eligibility criteria: chronic health conditions in ≥3 organ systems requiring subspecialist care and/or medical technology. Eligible caregivers were able to complete interviews in English, aged ≥18 years, and their child’s primary medical decision-makers. We excluded caregivers of CMC who 1) resided in long-term care facilities, and/or 2) were age <1 year, to ensure that participants had experience in caring for their child at home. We enrolled one caregiver per family unit to maximize breadth of responses.

We identified eligible caregivers from outpatient schedules and inpatient censuses of our institution’s Complex Care Center and Palliative and Supportive Care team. To minimize potential conflicts with clinical care, we excluded caregivers of CMC receiving clinical care from the interviewer (JY) at time of enrollment. A clinician known to the family (e.g., nurse) obtained permission for the research team to approach caregivers in-person and provide study details. All participants provided written informed consent prior to interviews and were compensated $35. We purposively sampled caregivers to attain diversity in socioeconomic status, duration of caregiving experience, and child’s medical conditions.

The semi-structured interview guide was developed by a multidisciplinary team with expertise in palliative care (JY, YS, AR), pediatric rehabilitation (AH), and clinical psychology (RN) and refined based on feed-back from three pilot interviews with CMC family caregivers. Open-ended questions elicited participants’ perspectives about key challenges of CMC caregiving, strengths and struggles in coping with caregiving-related stress, and their emotional well-being (see Supplement). A single male investigator (JY), with training in pediatrics, hospice and palliative care, and clinical research, conducted all interviews one-on-one, either in-person or via telephone (participant preference) until no new themes emerged (thematic saturation).^16^ Interviews were recorded, transcribed verbatim, and deidentified. Interviews were followed by a brief demographic survey collecting caregiver age, gender, race/ethnicity, presence and number of other household adults and children, receipt of home-health services, and child age, gender, medical technology use, medical conditions, and health insurance type (Table 1). As a proxy for socioeconomic status, we used the 2022 neighborhood-level Area Deprivation Index (ADI) which generates a composite measure of 17 indicators of social determinants of health using data from the American Community Survey and US Census Sur-vey.^17^ Participants were assigned a national percentile rank based on their residential census block group, with higher ADI percentiles indicating higher neighborhood-level disadvantage.

Two study team members (JY, ML) developed a preliminary codebook iteratively and inductively through line-by-line *in vivo* and descriptive coding and constant comparison on an initial subset of transcripts. The codebook was finalized through three rounds of concept and code review and revision with the multidisciplinary team (YS, AR, AH, RN). Over six cycles of coding and face-to-face comparison (via Zoom), three research team members (JY, ML, LM, and/or SW) independently applied the final codebook to each transcript and resolved any disagreements through group discussion and consensus.

Using human-centered design methodology (JY is a certified facilitator in LUMA *Institute’s System of Innovation* [a framework for conducting human-centered design]), the coding team then conducted a preliminary thematic analysis with an emphasis on identifying topics potentially novel and/or useful to CMC families, clinicians, researchers, and advocacy organizations.^18^ Human-centered design utilizes structured group-based activities which encourage equal participation and creative approaches to problem-solving to foster collaboration, innovation, and empathy. Through iterative group discussion with the larger multidisciplinary research team, we then defined and refined concepts into overarching themes and organized coding data accordingly. Lastly, our thematic analysis findings were presented to an existing community advisory board of CMC family caregivers and further refined based on their feedback. We used ATLAS.ti Web for data storage, coding, and analysis.

## Results

We identified, approached, and consented 28 of 29 eligible caregivers (one did not want to participate in research) between March 2022 and April 2023. We achieved thematic saturation after completing 19 interviews. Interviews were mainly conducted via telephone (14/19 [74%]) and lasted 49 minutes on average (range 37–66 minutes). Participants’ mean age was 43 (standard deviation [SD] 6.7) years and most identified as White (63%), female (90%), and biological or adoptive parents (100%) (Table 1). The average age of participants’ CMC was 10 years (SD 6.3; range 2–20 years). CMC experienced chronic health conditions most commonly affecting the neurologic (100%), gastrointestinal (100%), and respiratory (82.4%) organ systems. Every child utilized some form of medical technology (e.g., gastrostomy tube). Almost two-thirds of our sample lived in the most disadvantaged ADI quartile (Tables 2-4).

**Fig. 1.**
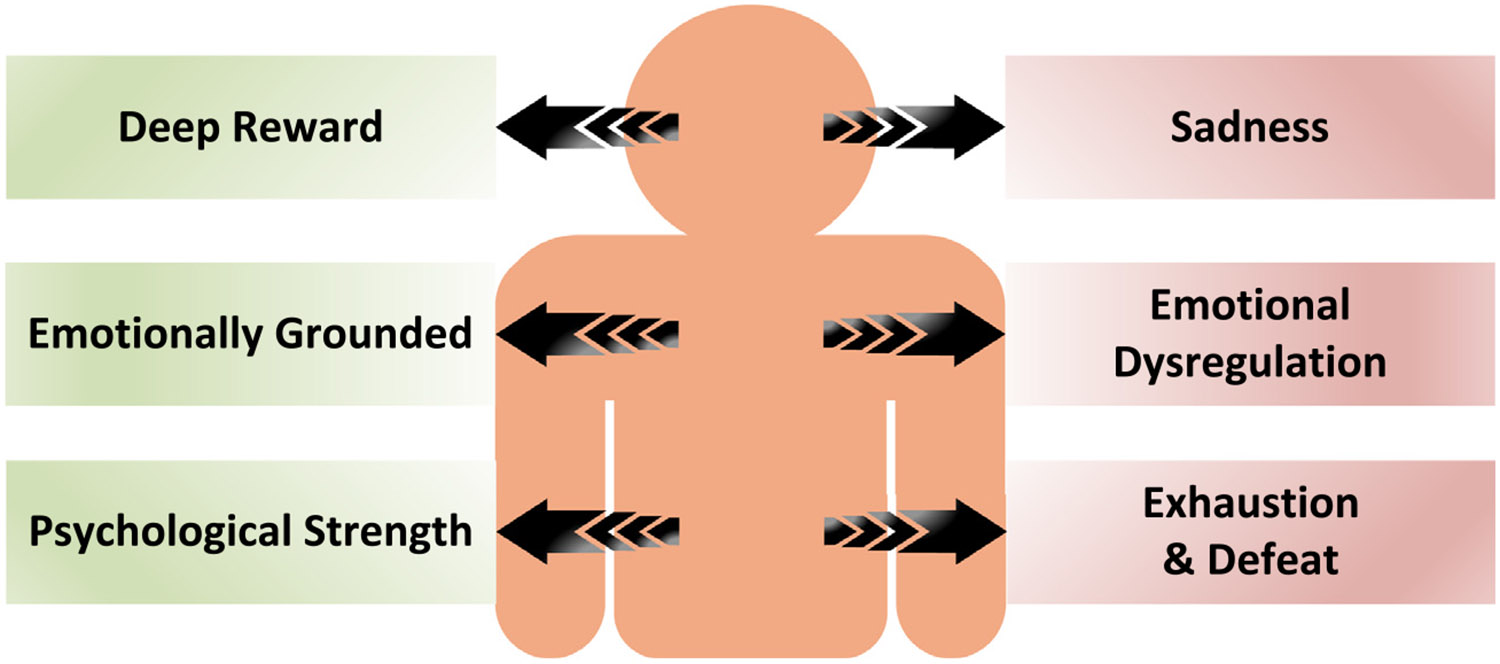
Positive and negative impacts on CMC caregiver emotional well-being.

Participants discussed that the intensity and unique challenges of CMC caregiving can spur personal growth, yet also adversely affect emotional well-being. We synthesized caregivers’ perspectives into three themes reflecting how their emotional well-being existed on a dynamic spectrum: 1) deep reward vs. sadness, 2) increased and decreased ability to control one’s emotions, and (3) psychological strength vs. exhaustion and defeat. As one mother put it, “I go back and forth with these feelings sometimes.” Themes and their conflicting nature are displayed in our conceptual model (Fig. 1).

### Theme 1: CMC caregivers experience deep reward and sadness

#### Deep Reward (n = 19/19).

Every participant discussed deriving personal fulfillment from an enhanced sense of purpose and a profound connection with their child. Caregivers found purpose in fully devoting themselves to their child’s health and quality of life. Reflecting on what she found most rewarding, one mother shared: “When people recognize that I am trying to give [child] the best life that she can have and that I’m taking care of her … When she seems really happy and smiley, that’s very rewarding because I know that in all her struggles, she’s still finding happiness.”

Participants also explained how the intensity of caregiving cultivated a uniquely strong bond with their child. One father stated, “the complexity kind of lends to a different kind of closeness and attachment.” This connection fostered a heightened sense of appreciation which enabled caregivers to love their child even more deeply. One mother explained what she wished clinicians would understand:

I don’t think they understand the beauty that comes along with caring for a complex child… I’m sorry, but they don’t completely understand how deep the love has to go in order for you to do the things you have to do day-to-day… The love that you have to have for a child like this is so much deeper.

#### Sadness (n = 19/19).

In contrast, participants also endorsed feelings of sadness over losing a life they had previously envisioned for themselves and their family. For example, caregivers discussed missing out on career opportunities and “typical” parenting mile-stones.

I just get really sad about how this life is different than if you had asked me 25 years ago… I had things that I wanted to do, and I don’t really get to do them. Having a baby girl, you think someday you’ll be going maybe to dance or sports’ practices with her, graduations, marriage, and children of her own. You grieve the loss of that.

Participants also focused on feelings of social isolation. Time constraints, concern about infections, and stigma around their child’s disabilities hampered engaging with the “outside world.” Friends and family gradually disappeared from their lives and participants found it difficult to connect with other parents. One mother explained why they stopped going to family’s birthday parties:

[When] I’ve taken him, he’s sitting in one of our arms on the couch. He’s not playing with the other kids. And above all else, nobody’s really paying attention to him. It’s harder to put yourself in that position than it is to just stay home where you’re going to give him one-on-one attention and love… So now we have to really think about, ‘Is this going to be worth it to bring him out? Is it going to trigger us? Is it going to almost make you a little bit resentful towards those friends and family?’

Some participants also reflected on their child’s life. Caregivers grieved the experiences and opportunities their child was deprived of (e.g., getting a driver’s license) and expressed feelings of guilt over their child’s perceived suffering:

I think sadness is definitely something to mention, sadness over his limits and what he’s missing out in life and that we may not have him for very long. There [are] definitely the emotions around watching him suffer every day, watching him have countless seizures and not be able to do anything about it, that is always very heart breaking for us.

### Theme 2: Caregivers report both enhanced and decreased ability to control their emotions

#### Emotionally Grounded (n = 19/19).

Every participant described being “grounded” by prior experiences and an enhanced ability to manage emotions. This did not mean caregivers no longer experience negative emotions, rather that they were better able to regulate their reactivity to stress, the intensity of their emotions, and recognize ruminative and/or unhelpful thoughts. One father shared:

Frustration often leads to a lot of the overthinking. And I’ve found that, when I’m getting frustrated, I need to take a little time for myself to wind down and kind of self-regulate and be a little more realistic about my expectations and knowing what I can control and what I can’t.

Participants added that how they perceived and interacted with other people had also positively changed. Caregivers described becoming more patient, forgiving, and willing to give others the benefit of the doubt. One mother stated, “You develop a level of patience that is far beyond anything you could ever imagine… [I am] more caring and patient with other people, whether it’s my family or doctors or whatever. So there’s lots of growing as a person.”

#### Emotional Dysregulation (n = 19/19).

At the same time, participants shared how unrelenting responsibilities, constant vigilance, and frequent setbacks resulted in persistent feelings of worry or being on-edge. “Doctors and nurses, they get to go home at the end of their shift and relax and, have a break… But for a parent, there is never that time off. It is a 24/7 worry that you can never escape from.”

Caregivers remarked that, over time, the worry could evolve into constant irritability and anger. Half of our participants discussed difficulty in controlling negative and intense emotions. One mother described her reason for seeking formal mental health treatment:

I felt all jittery inside. I was really impatient with everyone around me. I was very emotional and wanted to cry over everything. And I would get physically sick over different situations in our life that were happening as they came up.

### Theme 3: CMC caregivers feel both psychologically stronger and exhausted and defeated

#### Psychological Strength (n = 18/19).

Almost all participants discussed how their self-confidence grew as they learned to manage their child’s care and that this confidence carried over into other aspects of life. Caregivers felt more capable in tackling other life challenges and self-assured in social situations. One participant reflected, “I think it’s just made me more confident… as a person, in my skill set, and my career… just confident in all areas.”

Participants also explicitly discussed that living through setbacks and their child’s ups and downs had made them psychologically stronger. Caregivers were better equipped to tolerate uncertainty and unpredictability and “power through” adversity, often by drawing motivation from their child. One mother explained, “It has made me a stronger person. You have to look at things and you realize that you can’t change this, you can’t change that. This may happen, that may happen. You finally figure out and understand that you’re not always going to have a play in the book.”

#### Exhaustion & Defeat (n = 15/19).

Participants simultaneously discussed the toll caregiving had taken. Intense and unending demands left many caregivers physically, cognitively, and emotionally exhausted. One mother remarked:

I’ve been in survival mode just doing whatever needs done. And with my daughter, it has not stopped. We find ourselves saying, ‘When is it going to end?’ And we know that it’s not going to end. We want it to slow down, but every time it seems like it’s slowing down, it seems to pick right back up… The constant survival mode - it really plays on you emotionally and physically.

Participants also remarked how the build-up of setbacks could culminate in feelings of powerlessness. One father explained, “It’s all long-term frustration and defeat. I think, more than anything, feeling so helpless that you’re not able to figure out the problem because, as parents, we want to fix our children, right? We want to make sure they’re safe and everything’s okay. That’s our job.”

For some, these feelings manifested as pessimism about the future. One mother commented, “People will ask how she is doing. And I’m like, ‘Well, she’s good for now. I don’t know for how long.’ I’m thinking in my head, ‘It could be two weeks, and we’ll be back in the hospital.’ …It’s a kind of pessimism I can get myself into.”

## Discussion

In this qualitative study, we aimed to describe CMC family caregivers’ perspectives about their emotional well-being in a balanced and holistic manner, capturing tensions and fluidity. Three themes emerged: 1) deep reward vs. sadness, 2) increased vs. decreased emotional regulation ability, and 3) psychological strength vs. exhaustion and defeat. Notably, seemingly conflicting experiences could coexist. CMC caregivers reported experiencing a range of intense emotions, often at opposite ends of a spectrum, either concurrently or oscillating between them.

Our findings regarding the adverse aspects of caregiver emotional well-being are consistent with prior research describing the experiences of parents of children with serious illness. For example, Teicher et al.’s recent study among parents of CMC in Canada also identified feelings of sadness about missing out on life experiences, social isolation, and their child’s uncertain prognosis and symptom burden.^6^ Similarly, our participants’ discussion about constant worry, emotional lability, and exhaustion and defeat stemming from their child’s fragility and demanding around-the-clock responsibilities, aligns with multiple qualitative studies among parents of children with chronic life-limiting conditions,^19-21^ and survey-based work in which CMC family caregivers reported clinically significant symptoms of anxiety and sleep-related impairment, as well as significantly reduced emotional regulation ability.^22^ That these themes also mirror findings well-documented among other high-intensity caregiver populations (e.g., caregivers of adults with dementia or advanced cancer) suggests existing evidence-based interventions might be well-suited for adaptation to CMC family caregivers.^23^ Interventions such as peer navigation, in which caregivers are connected to community members who have similar caregiving experiences and undergone training in providing emotional support and advocacy, are supported by a growing body of research.^24^ Similarly, psychoeducational interventions which teach caregivers skills based in cognitive-behavioral therapy and mindfulness-based stress reduction are promising as well.^25^

By including both a strengths-based and hardship-focused lens in our study’s analysis, we build on the CMC family caregiver literature in several ways. First, we demonstrate that CMC caregivers experience a dynamic range of intense and seemingly opposing emotions. Despite their role’s inherent challenges and setbacks, our participants reported strongly positive perceptions about the impact of caregiving on their emotional well-being. This is consistent with our work in which CMC caregivers reported psychological strengths (e.g., meaning and purpose, self-efficacy) at or above US population norms while also reporting elevated levels of emotional distress.^22^ It also echoes core themes from prior research on parental hope: 1) parents of seriously ill children can simultaneously hold hope for the future and accept clinical realities, and 2) the focus of and ease in which parents can access their hope often changes.^26,27^ Our participants’ discussions were characterized by a similar tension and fluidity; sometimes CMC caregivers find it easy to live in a positive space, other times it is difficult not to dwell on adversity.

We also better describe the positive changes to emotional well-being most salient to CMC caregivers. In our *Deep Reward* theme, participants highlighted their sense of purpose and connection with their child. These concepts align with work exploring existential concerns such as meaning-making, spiritual well-being, and “good parent” ideals.^28-31^ Among caregivers of adults, a growing body of evidence demonstrates that supporting caregivers’ existential well-being (e.g., sense of meaning) is associated with decreased emotional distress and caregiver burden.^32^ Similarly, enhanced emotional grounding and psychological strength align with positive changes well-documented in the broader caregiving literature. Namely, these findings echo work among caregivers of both adults and children with cancer demonstrating personal growth in the domains of emotional regulation, self-efficacy, and resilience; central aspects of emotion- and problem-focused stress-coping ability.^33,34^

This work has important clinical and research implications. For front-line CMC clinicians, our findings should serve as a reminder that 1) factors beyond a child’s medical or developmental outcomes shape caregiver emotional well-being, and 2) caregivers constantly manage a complex and often conflicting array of emotions. CMC caregivers do not view themselves as downtrodden and hopeless, nor as indefatigable superheroes who always remain calm and collected. Our field can take the lead in modeling humanistic and holistic approaches in supporting CMC families. This starts with the conscious application of patience and demonstration of empathy (i.e., recognizing and responding to emotions) in interacting with CMC families.^35^ We also urge clinicians to consider intentionally validating caregivers’ efforts and positive sentiments as a targeted strategy to bolster therapeutic alliance and caregiver emotional well-being.^36^ CMC clinicians can also incorporate informal check-ins (“Can I take a moment to ask how *you* have been feeling?”) and formal screening for emotional distress (e.g., PROMIS short-form measures of anxiety and/or depression)^37^ into routine clinical workflows, in addition to developing protocols to connect distressed caregivers with appropriate mental health supports.^38^ Lastly, given the high rates of socioeconomic adversity experience by CMC,^12^ our participants’ descriptions of defeat and exhaustion are further evidence that structural reforms are critically needed. In addition to strengthening the home-health workforce, clinician leaders and family organizations must continue to advocate for increased investments in paid family caregiving models, community-based supports, and respite services.^39-41^

From a research perspective, communication scientists should consider using our findings to enhance current shared decision-making frameworks.^42,43^ For example, instead of limiting conversations about life-sustaining medical decisions to a child’s risk of mortality and/or neurodevelopmental outcomes, balanced and nonjudgemental discussions about how a specific treatment (e.g., tracheostomy) might impact caregivers’ well-being could be included as well.^44^ Our themes regarding reward, emotional regulation, and psychological strength could also guide selection of positively oriented family-centered outcomes and therapeutic targets for future CMC-related research.^14^ For example, investigators might consider tracking caregivers’ sense of meaning and purpose over time to identify timepoints when and/or subgroups for whom strengths-based psychosocial interventions might be particularly helpful. Alternatively, interventionists could consider adapting and testing behavioral interventions, already shown to enhance emotional regulation in other caregiver populations, among CMC family caregivers.^23,45,46^

This study has several limitations. It was conducted among a small group of caregivers at a single, urban, academic children’s hospital that houses a Complex Care Center able to provide comprehensive and multidisciplinary CMC-focused outpatient services, which limits our findings’ generalizability. Specifically, our participants’ perspectives about caregiving’s positive impacts to their emotional well-being might not apply to caregivers of CMC with reduced access (e.g., distance and transportation challenges) to well-resourced institutions and/or live in settings with limited home- and community-based supports and services. Relatedly, our participants might have been well-situated to discuss and emphasize caregiving’s positive effects as they had 10 years of caregiving experience on average. Though we successfully recruited a racially and socio-economically diverse sample, our participants primarily identified as mothers and were English-speaking, preventing our ability to explore differences by gender and language groups. We also note that the ADI is an imperfect proxy for participants’ socioeconomic status as it reflects the average conditions of a neighborhood and cannot fully account for the variance of individuals’ personal circumstances within neighborhoods. Lastly, as participants only completed one interview, findings regarding the longitudinal evolution of caregiving’s impacts are vulnerable to recall bias.

In summary, caring for CMC affects family caregivers’ emotional well-being in multiple and dynamic ways. Importantly, caregivers often experience seemingly conflicting effects concurrently. These findings can offer guidance for clinicians caring for these families and inform the development of future psychosocial support interventions for CMC family caregivers.

## Supplementary Material

1

Supplementary material associated with this article can be found in the online version at doi:10.1016/j.jpainsymman.2025.03.028.

## Figures and Tables

**Table 1:** Participant and Child Characteristics (*n* = 19)

Caregiver Characteristics	N (%)
Mean Age (years)	43 (SD 6.7)
Female Gender	17 (89.5)
Race/Ethnicity	
Black	4 (21.2)
Hispanic	1 (5.3)
Multi-Racial	1 (5.3)
White	14 (73.6)
Area Deprivation Index Percentile	
≤25	1 (5)
26–50	2 (11)
51–75	4 (21)
>75	12 (63)
Duration of Caregiving Experience (years)	10.6 (SD 6.3)
Other Adult(s) Living in Household able to Help with CMC Care	13 (68.4)
Other Children Living in Household	11 (57.9)
Receives Home-Health Support (e.g., home nursing)	11 (57.9)
Child Characteristics	N (%)
Mean Age (years)	10.8 (SD 6.3)
Female Gender	10 (52.6)
Organ Systems Affected by Chronic Medical Condition	
Cardiovascular	8 (42)
Congenital/Genetic	13 (68)
Gastrointestinal	19 (100)
Hematologic or Immunologic	9 (47)
Metabolic	5 (26)
Neurologic	19 (100)
Prematurity/Neonatal	9 (47)
Renal/Urologic	8 (42)
Respiratory	16 (84)
Utilizes Medical Technology	19 (100)
Gastrostomy	18 (95)
Oxygen	11 (58)
Mechanical Ventilation	7 (37)
Central Line	4 (21)
Implanted Electrical Device (e.g., vagal nerve stimulator, intrathecal pump)	8 (42)
Wheelchair	16 (84)
Health Insurance Type	
Public	9 (47)
Private	2 (11)
Both Public and Private	8 (42)

**Table 2:** Illustrative Quotes for Theme 1: CMC Caregivers Experience Deep Reward and Sadness

Deep Reward	Theme 1 Subthemes		Sadness
“It’s taught me how much I can love… Whenever you’re young and people tell you, ‘Oh, you don’t know how much you’ll love that baby,’ and you’ll think, ‘Well, yes. I can kind of guess I’ll love this baby a lot.’ But then, you don’t know just how much. I almost feel like she’s an extension of me because I do so much for her and have to be her advocate.” “I think that there is that deeper level of connection, where she’s nonverbal, and she’s not able to communicate or tell me like, ‘Hey, I’ve never heard, mom, I love you or thank you or any of that.’ But I think in knowing that all the decisions I make I feel are in her best interest…. I feel like I have a closer connection to her. It’s hard to explain because I’m very close with my other kids, but I feel that her medical condition and how much she relies on me, has kept that relationship closer.” “You just appreciate things a lot more. And you pick what’s important in life. Like, is fluffing my pillows more important? I used to be so anal about things at my house. And now they’re just sitting on my table. You just find value in life caring for someone– that’s hard. If you haven’t experienced it, it’s hard for somebody to understand it. It takes a tremendous amount of love to give so much to one being and one person in order for them to succeed.”		“Sometimes you’re just really sad and it’s weird to be actually my age and you just feel sad. You feel like life is just really going on around you. You can’t really get in it ever. Maybe for short little snippets. I’m sure other caregivers experience this, you can just feel sad alone and just kind of just so oddball.” “I have so much guilt about so many things which she can and can’t do. And I hate people putting limitations on her. I hate that. I was getting her a service dog… to bring her some kind of joy. We bought her a puppy just two weeks ago. And when I mentioned it with the infectious disease [doctors] last week– I mean, their faces got crazy. And I was like, ‘What?’ And they said that if she was to get her skin broken– you know how they nip and bite when they’re puppies, that it’s not like us, we can’t just wash it off, I have to take her to the ER to get antibiotic treatment. And then [doctor] said he was so sorry that they took that little bit of joy I had.” “Like family reunions and different family functions, and it’s like– especially now I want to participate, but then it’s just so much getting [child] there and then with all– everything with her respiratory and all this different stuff, it’s better just to stay close to home. So I miss out on a lot and then I get in the slumps because I want to be out still living life and being with family, but it’s like I can’t.”	

**Table 3:** Illustrative Quotes for Theme 2: CMC Caregivers Report both Enhanced and Decreased Ability to Control their Emotions

Emotionally Grounded	Theme 2 Subthemes		Emotional Dysregulation
“I feel more balanced now. I don’t let the little things bother me. I don’t worry about dusting my windowsills. I used to worry about my house being really clean when people came over. I don’t care. Doesn’t matter. I’m getting through the day. That’s all that matters. And my kids are healthy and doing good. So I think it really helped me just get rid of a lot of petty things– or little things that I used to worry about.” “I feel like through the years; I’ve learned to just be a little bit more forgiving and flexible with things, knowing, time after time, that things are just going to play out how they’re going to play out. [I realize], that’s a hard pill to swallow, and it’s easier said than done.		“You’re hypersensitive. I literally can remember hearing a fly flying through my bedroom while I was sleeping because I’m listening. So it is a constant on… There is no off. There’s no stop… So it’s hard sometimes because you just can’t seem to– you can’t pull your words together. You can’t roll in those emotions and set them back like I normally would.” “The biggest thing I’ve noticed is my inability to control my emotions at this point, which I’ve always been very in control of everything, and now I have no control. And then that upsets me… It’s really hard to accept that you’re not handling things the way you used to.”	

**Table 4:** Illustrative Quotes for Theme 3: CMC Caregivers Feel both Psychologically Stronger and Exhausted and Defeated

Psychological Strength	Theme 3 Subthemes		Exhaustion & Defeat
“It’s been a learning and growing experience for me because I never thought I would be able to do this. But now, I’m doing it and it’s not a problem because this is my child and I just do it. It’s a sense of accomplishment at times because once again, this is something that I never thought I would ever have to do… Now it’s like I’m wanting and willing to do whatever it takes.” “When it comes to caring for my son, I’ve pretty much convinced myself, ‘This is easy. I’ve had it way harder. I can do this. This is not going to be a problem at all. I’ve been through more difficult situations.’ …You have a kid and you’re like, ‘Oh, no. Can I do it?’ Well, now, I’m like, ‘Yeah. I can do this. This is not a problem. I’ve got this.’” “Just to see how hard it is for her and seeing how she still has that fight in her to just keep going and still have a smile on her face. And amongst all of the pain that she has, one of the big things is like, ‘if she could do it, we can too.’”		“I just feel emotionally exhausted or worn out, partially from watching him go through whatever he went through and not being able to help him as his mom, partially because the emotional part of being in the hospital where I’m making decisions for him and trying to advocate for him and fighting for him to get back to where he needs to be or where I would like him to be.” “I might not show the emotion of someone that’s never dealt with a sick person that other, if you’re not in medical field, you might show. I just tell them how strongly concerned I was and I expected that they heard me and they would take me into consideration. But I’ve had doctors say, ‘Well, you don’t seem that concerned.’ …I came in, I told you what was going on. If there’s requirements that I cry or scream or come 500 times when you said, ’This is what we’re doing. This is all we’re doing,’ you got to let me know that.” One mother describing her challenges with obtaining padded heel cups for her child’s pressure ulcer: “It’s to a point now where I’ve given up. [After the last admission] they wrote a script to the supply company, but the company calls me a week later and says, ‘Oh, we can’t fill this because it’s not written correctly,’ and all this stuff. And I’m just like, ‘You know what? I’m done.’ So now [child] has a boot and we just use foam padding… I just gave up with that. It’s something little, but it frustrates the heck out of me.”	

## Key Message

We describe the perspectives of family caregivers of children with medical complexity (CMC) about their emotional well-being. CMC family caregivers report positive and negative emotions which are often in tension and dynamic. Themes included reward vs. sadness, increased vs. decreased emotional regulation ability, and psychological strength vs. exhaustion and defeat.

## Appendix

### Interview Guide

My name is _____. Thank you so much for agreeing to participate. I will ask you a series of questions about your experiences in caring for your child, sources of stress related to this caregiving, and how you deal with this stress. We are hoping to learn if there are additional ways in which we could offer services that would better support you in caring for your child.

There is no right/wrong answer to these questions, you are the expert. All your responses will be confidential and will not be shared with anyone from your child’s health care team unless we learn that you or someone you are involved with is in serious danger. This interview will in no way affect your child’s health-care. If you feel uncomfortable at any point, we can pause the interview and you do not have to continue if you wish to stop. Similarly, you do not have to answer a question if you would prefer not to. Lastly, we are going to audio-record and transcribe this conversation for reference.

***State ID & DATE at start of recording***

Contributors to stress

1. To start, I would like you to think about what a normal day caring for your child involves. What would you want your child’s doctors and nurses to know about what it is like?
  - Probes:
    - How has caring for a child with complex health conditions been different than what you were expecting? …. How has caring for [CHILD] been different than caring for your other children?
    - Tell me about the parts of your day that are not directly focused on your child.
  - Follow-up:
    - How has caring for [CHILD] changed over time? Do you think about your experiences in different phases/stages?
2. Could you tell me about a time, related to caring for your child, that stands out as being particularly stressful?
  - Probes:
    - Tell me more about what made this experience so challenging?
  - Follow-up:
    - What else makes you feel stressed?
    - Anything not directly related to caring for your child?
3. Can you describe the emotions you typically feel when challenges like [the one you described] come up?
  - Probes:
    - For example, some parents have talked about how they often experience anger when things like insurance claims are denied. Alternatively, some have mentioned feeling helpless or overwhelmed if their child develops a new issue. What has your experience been like?
4. What have you found to be helpful in dealing with the stress of challenges that you have described?
  - Probes:
    - When you’re feeling stressed out, what do you do to cope?
  - Follow-up:
    - When you say [XXX], tell me more about what that involves?
5. Parents have told us that the type of challenges you have described negatively impacts their mental health. What has your experience been like?
  - Probes:
    - For example, some people have talked about constantly feeling anxious. Others have mentioned having difficulty controlling their anger or having trouble falling asleep.
  - Follow-up:
    - Some parents have also mentioned seeing their own therapist, counselor, or psychologist. If you have done this, what has your experience been like?
    - Similarly, could you tell me about your experiences taking medications for your mood. For example, some people take medications for depression or for anxiety.
6. What don’t others understand about the positive parts of caring for your child that’s different from caring for children who don’t have complex health conditions?
7. What has been most rewarding about caring for your child?
  - Probes:
    - What about your role as your child’s caregiver is most meaningful?
  - Follow-up:
    - Has this changed over time?
8. How has having a child with complex health conditions positively impacted your emotional well-being?
  - Probes:
    - For example, some parents have said they are more confident in themselves. Others have remarked about developing a new sense of purpose. Tell me about how caring for your child has changed you positively?
    - Could you tell me about a time that stands out in which you were particularly happy? Or proud?
9. Knowing what you know now, what advice would you have given a younger version of yourself about dealing with the stress and challenges that come up in caring for your child?
  - Probe:
    - What advice would you give to new parents of children with complex health conditions?
10. Some parents have mentioned that caring for a child with complex health conditions can be really isolating. What has your experience been like?
11. Who in your life do you turn to for emotional support? This could be a specific person or a community like a church.
  - Follow-up:
    - Can you tell me more about the support they provide?

Thank you so much for taking the time to do this interview! I’m going to pause and ask you to complete the quick series of multiple choice questions so we can better describe who participated in the interviews.

## Abbreviations:

CMC: children with medical complexity
SD: standard deviation
